# Prescriptive variability of drugs by general practitioners

**DOI:** 10.1371/journal.pone.0189599

**Published:** 2018-02-20

**Authors:** Magda Bucholc, Maurice O’Kane, Siobhan Ashe, KongFatt Wong-Lin

**Affiliations:** 1 Intelligent Systems Research Centre, University of Ulster, Magee Campus, Londonderry, Northern Ireland, United Kingdom; 2 Altnagelvin Area Hospital, Western Health and Social Care Trust, Glenshane Road, Londonderry, Northern Ireland, United Kingdom; University of Oxford, UNITED KINGDOM

## Abstract

Prescription drug spending is growing faster than any other sector of healthcare. However, very little is known about patterns of prescribing and cost of prescribing between general practices. In this study, we examined variation in prescription rates and prescription costs through time for 55 GP surgeries in Northern Ireland Western Health and Social Care Trust. Temporal changes in variability of prescribing rates and costs were assessed using the Mann–Kendall test. Outlier practices contributing to between practice variation in prescribing rates were identified with the interquartile range outlier detection method. The relationship between rates and cost of prescribing was explored with Spearman's statistics. The differences in variability and mean number of prescribing rates associated with the practice setting and socioeconomic deprivation were tested using t-test and *F*-test respectively. The largest between-practice difference in prescribing rates was observed for Apr-Jun 2015, with the number of prescriptions ranging from 3.34 to 8.36 per patient. We showed that practices with outlier prescribing rates greatly contributed to between-practice variability. The largest difference in prescribing costs was reported for Apr-Jun 2014, with the prescription cost per patient ranging from £26.4 to £64.5. In addition, the temporal changes in variability of prescribing rates and costs were shown to undergo an upward trend. We demonstrated that practice setting and socio-economic deprivation accounted for some of the between-practice variation in prescribing. Rural practices had higher between practice variability than urban practices at all time points. Practices situated in more deprived areas had higher prescribing rates but lower variability than those located in less deprived areas. Further analysis is recommended to assess if variation in prescribing can be explained by demographic characteristics of patient population and practice features. Identification of other factors contributing to prescribing variability can help us better address potential inappropriateness of prescribing.

## Introduction

In recent years, NHS spending on drugs has substantially risen, from £13.0 billion in 2010/11 to £16.8 billion in 2015/16 [1]. Most of the expenditure on prescribed medicines is incurred in primary care and closely related to the steadily growing workload of general practitioners (GPs) [1]. In England, patient consultations with GPs increased by 16% in the period 2007–14 [2] whereas in Northern Ireland, the GPs workload grew by 22% over the same period [3]. In addition, there has been an approximately 60% increase in prescription items dispensed from 2005 to 2014 in Northern Ireland [3] and a corresponding 50.4% rise in the number of prescriptions dispensed in England [4].

The National Audit Office report found that substantial savings for the NHS could be achieved by improving the overall quality and cost-effectiveness of prescribing [5]. Accordingly, a lot of interest has been focused on variation in prescribing practice as a potential source to save money [5,6]. Despite a wealth of literature on prescribing patterns [5–9], there is a lack of full understanding of factors that contribute to between-practice differences in prescribing. Among key influences upon prescribing variation, the demographic and socio-economic characteristics of patient population (e.g. age, ethnicity, deprivation) are most often acknowledged by researchers [10,11]. GP practices with a greater proportion of people in older age groups were more likely to prescribe minor tranquilisers [10], sex hormones, anticoagulants and protamine, and treatments for glaucoma [12]. Significant differences in prescribing were also associated with the level of deprivation [13]. Several studies have shown that extent of local deprivation influences antidepressant and lipid-lowering medication prescribing [14–16]. On the other hand, lower volume of prescribing was observed in practices with higher proportions of patients from ethnic minority populations [17]. Practice features were also among factors contributing to the variation in prescribing behaviour. Examples of that include higher prescription rates issued by practices located in urban areas with a greater proportion of female GPs [18]. Lower prescribing was found for single-handed practices, practices in rural areas, with a higher average age of general practitioners, and with GPs born outside the UK [15,19,20].

Differences in characteristics of GP practices or a patient population do not always explain GPs prescribing behaviour. In many cases, the variability in prescribing rates is associated with inefficient or inappropriate prescribing [5,21]. It has been estimated that the prescription costs could be reduced by as much as £1bn if unwarranted variations in prescribing levels were eliminated and the drugs were prescribed with the same standard [21]. Better efficiency and appropriateness in prescribing practice could be achieved by addressing the over- or under-utilisation of drugs. It was shown that prescribed medications are often taken for long periods beyond the point when they are needed and around 30% of drugs are abandoned by patients [22–25]. Major NHS savings could also be generated by using treatments that are most cost-effective. Moon et al. [26] showed that a large number of GPs are still prescribing brand name medications, even though the cheaper, equally safe and effective alternatives are available.

The aim of this study was to investigate temporal changes in rates and costs of prescribing as well as between-practice variation in prescribing. In addition, we examined if prescribing behaviour of GPs was related to the practice setting and socioeconomic deprivation.

## Materials and methods

### Data and pre-processing

We analysed the number and actual cost of prescription items issued by 55 general practices within the Northern Ireland Western Health and Social Care Trust (WHSCT) during twelve consecutive periods of 3 months, starting from Apr 2013 to Mar 2016. The actual cost of prescriptions was defined as the estimated cost to the NHS calculated by subtracting the discount per item from the gross cost which is the basic price of a drug.

The GP prescribing data was obtained from the Business Services Organisation’s (BSO) prescribing and dispensing information systems [27]. It includes prescribing for all GPs and other non-medical prescribers who are attached to GP practices i.e. nurses, pharmacists, optometrists, chiropodists, and radiographers. To allow temporal comparison of prescribing data, the number of drug prescriptions and their total cost calculated for each general practice was adjusted for the total number of patients in each practice and expressed as prescriptions/cost (£) per patient.

Given data from the Census Office of the Northern Ireland Statistics and Research Agency [28], a practice was designated as urban if its postal address was situated in a settlement of more than 10,000 residents. Under this definition, 31 practices were categorised as urban and 24 as rural.

In addition, practices were categorised based on the Northern Ireland Multiple Deprivation Measure (NIMDM) at the level of Super Output Area (SOA) [29]. The NIMDM consists of seven domains i.e. Income; Employment; Health, Deprivation, and Disability; Education, Skills and Training; Proximity to Services; Living Environment; and Crime and Disorder. On this overall measure, the SOA with a NIMDM rank of 1 is considered the most deprived, and 890 the least deprived. Accordingly, a practice situated in SOA with the NIMDM rank larger than 445 was designated as ‘located in a less deprived area’ while a practice situated in SOA with a NIMDM rank smaller than 445 was designated as ‘located in a more deprived area’. Under this definition, we identified 11 practices ‘located in less deprived areas’ and 44 practices ‘located in more deprived areas’.

### Statistical analysis

The variation in the number and cost of prescriptions per patient was assessed by calculating the variance (σ^2^) for each of the 12 considered time points [30]. In addition, we analysed changes in mean (μ) and range of the rate and cost of prescriptions.

The outlier GP practices were identified for all time points using the interquartile range (IQR) method for outlier detection [31]. Accordingly, a practice with the prescribing rate that fell outside either 1.5 times the IQR below the first quartile or 1.5 times the IQR above the third quartile, was considered to be an ‘outlier’. We however acknowledge that a statistical outlier in terms of prescribing rate is not necessarily an example of inappropriate practice.

The differences in the mean number of prescribing rates, for the rural and urban practices as well as practices located in areas of different levels of socioeconomic deprivation, were assessed using an unpaired t-test [31]. The equality of variances of prescribing rates for above-mentioned practice categories was evaluated using F-test [30]. The normality of prescribing data was confirmed with Shapiro-Wilks test [32].

To determine if temporal changes in variability of rates and costs of prescribing underwent a statistically significant upward or downward trend over the study period, we used the Mann–Kendall test which has been commonly employed to detect trends in series of data [33,34].

The relationship between rates and cost of prescribing was explored with Spearman's rank correlation (*rho*) [35]. We chose the Spearman correlation measure due to it insensitivity to individual contribution of outliers. The strength of correlation was defined as very weak for |*rho*| = 0.2 to 0.39, moderate for |*rho*| = 0.4 to 0.59, strong for |*rho*| = 0.6 to 0.79, and very strong for |*rho*| = 0.8 to 1 [35].

## Results

The total number of patients registered at 55 general practices providing services throughout 2013–16 increased from 318,057 in 2013–14 to 326,429 in 2015–16. Over this time, the total actual prescription cost continued to rise from £58,669,971 in 2013–14 to £63,803,168 in 2015–16.

**Fig 1:**
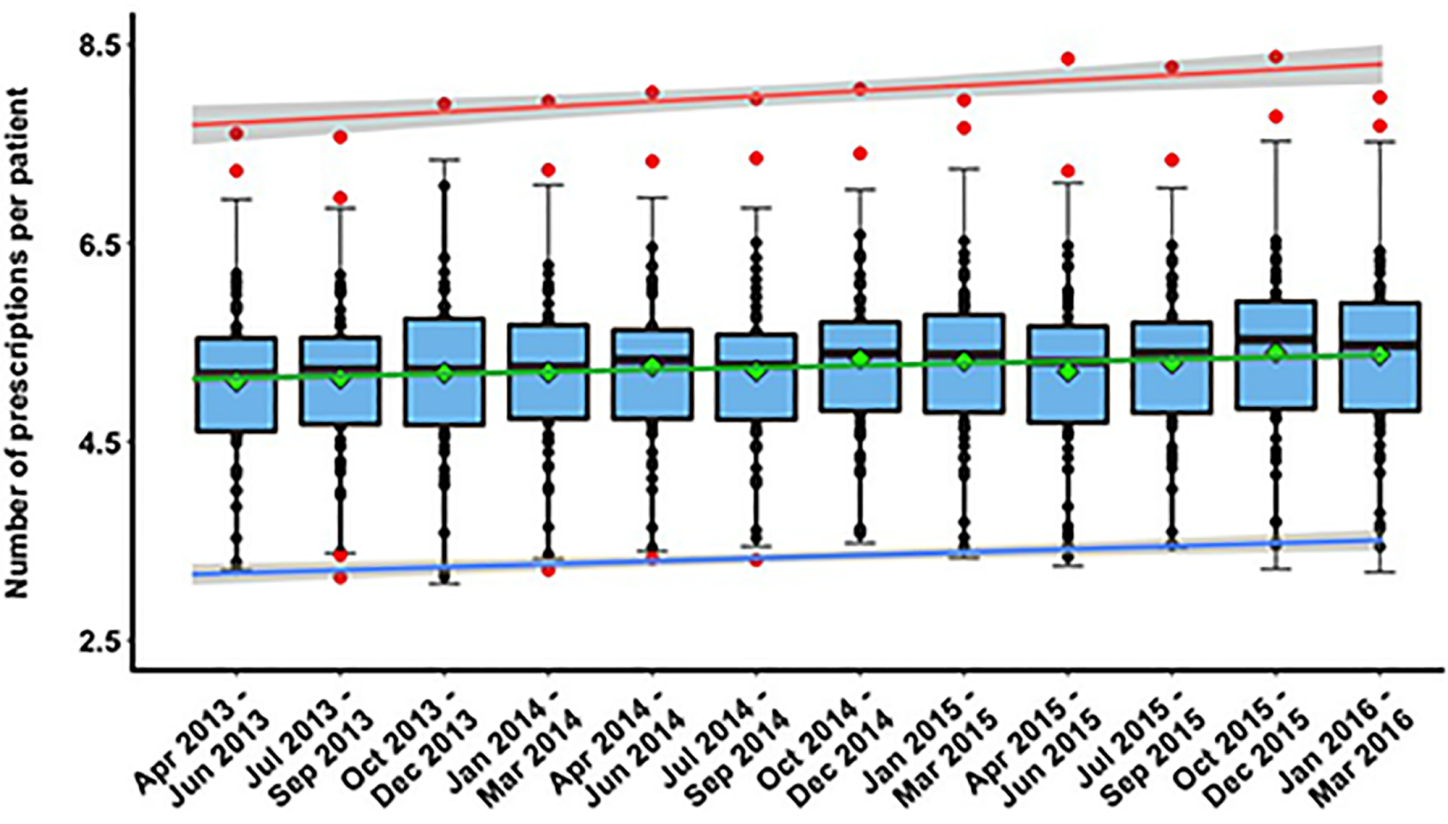
Temporal variability in the standardized number of prescriptions. Each data point (dot): a single practice. Solid, horizontal line inside the box: median of data. Green diamond: mean. Lower and upper "hinges” of the boxplots: 1^st^ and 3^rd^ quartiles, respectively. Red, green, and blue lines: trend lines for maximum, average, and minimum values of prescription rates respectively. Lower and upper extremes of whiskers: interval boundaries of the non-outliers (black dots). Data outside interval (red dots): outliers.

Fig 1 shows the magnitude and temporal changes in variability of the number of prescriptions per patient. We observed pronounced differences in drug prescribing rates among individual practices. The largest between-practice difference in prescribing rates was observed for the quarter of Apr-Jun 2015, with the number of prescriptions ranging from 3.34 to 8.36 per patient. During this period, the prescription rate for the practice with the largest number of prescriptions per patient was ~ 60% higher than the average prescribing rate for all the practices (μ = 5.20, 95%CI = [4.96,5.44] prescriptions per patient). The smallest between-practice difference in prescribing rates was observed in the period Apr-Jun 2013, with the number of prescriptions ranging from 3.21 to 7.60 per patient. At that time, the practice with the highest prescribing rate issued ~ 49% more prescriptions per patient compared to the average prescribing rate of μ = 5.11, 95%CI = [4.89, 5.33]. The high inter-practice variability in drug prescribing behaviour was caused by: 1.8% (Oct-Dec 2013), 3.6% (Apr-Jun 2013, Oct 2014 -Mar 2016), 5.5% (Jan-Sep 2014), and 7.3% (Jul-Sep 2013) of GP practices with outlier prescribing rates. By eliminating the effect of these outliers (i.e. practices with higher or lower prescribing rates than the calculated outlier cut-off values), we were able to reduce the between-practice variability in prescribing rates from 21% (σ^2^ reduced from 0.71 to 0.59 in Oct-Dec 2013) up to 70% (σ^2^ reduced from 0.67 to 0.39 in Jul-Sep 2013) (S1 Table). It is worth highlighting that despite varying number of outliers identified in each quarterly period, they were mostly the same practices: one practice (with substantially higher prescribing rate than outlier cut-off values) was identified as an ‘outlier’ throughout the studied period while two other practices (one with higher and the other with lower prescribing rate than outlier cut-off values) were labelled as ‘outliers’ at 11 and 4 considered time periods respectively.

**Fig 2:**
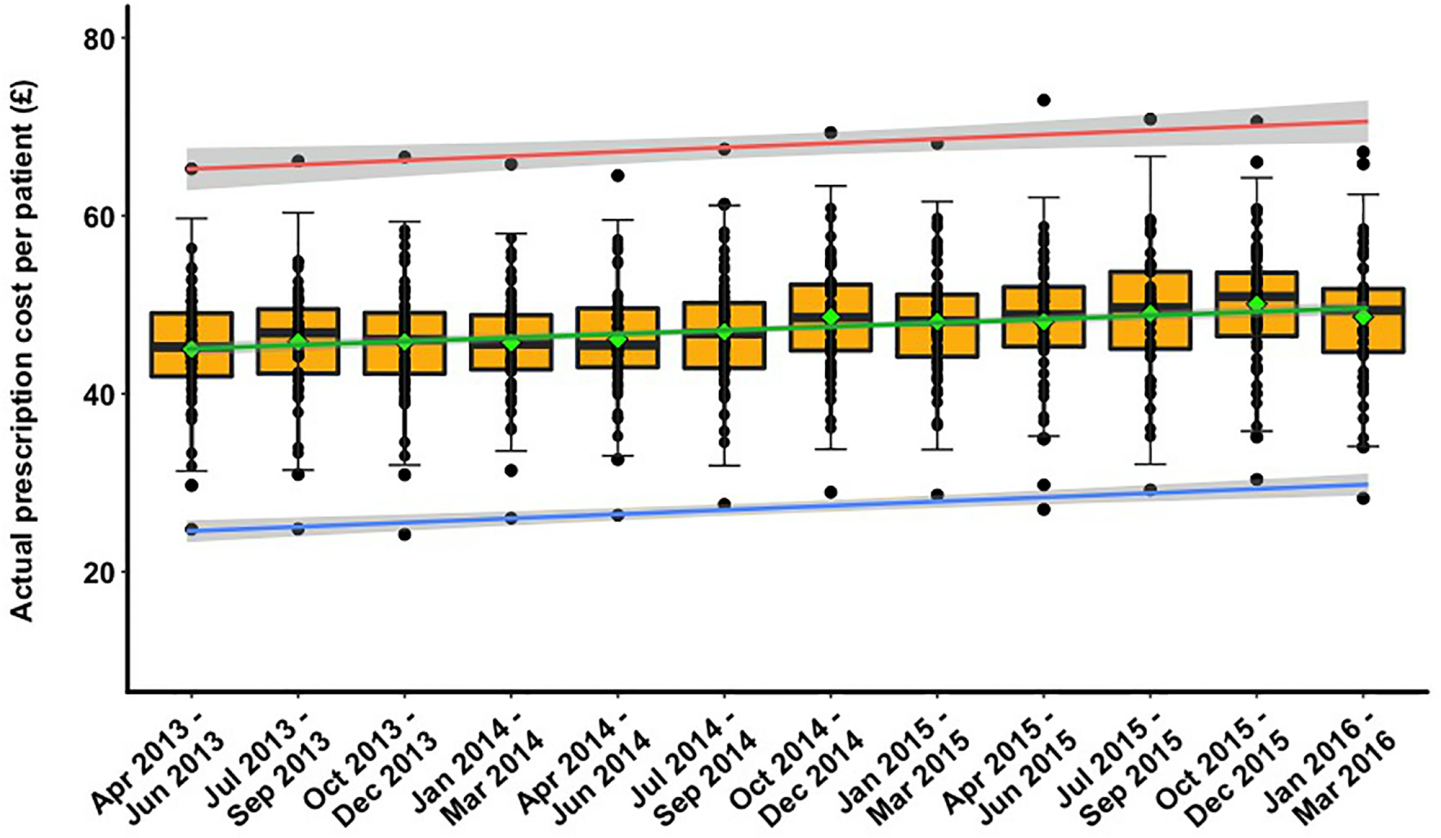
Temporal variability in the actual cost of prescribed medications per patient. Each data point (dot): a single practice. Solid, horizontal line inside the box: median of data. Green diamond: mean. Lower and upper "hinges” of the boxplots: 1^st^ and 3^rd^ quartiles, respectively. Red, green, and blue lines: trend lines for maximum, average, and minimum values of prescription costs respectively.

Temporal variability in the actual cost of prescribed medications per patient is shown in Fig 2. The largest between-practice difference in prescribing costs was observed for the quarter of Apr-Jun 2014, with the prescription cost per patient ranging from £26.4 to £64.5. During this time period, the highest actual cost of prescribed medications per patient for the individual practice was ~40% higher than the average prescribing cost of μ = £46.1, 95%CI = [£45.2, £47.0]. In addition, the average cost of prescribing per person was observed to increase by 11.3%, 95%CI = [10.4%,12.2%] over the period of investigation; from £45, 95%CI = [£43.2, £46.8] in the first quarter (Apr-Jun 2013) to £48.6, 95%CI = [£46.7, £50.6] in the last quarter (Jan-Mar 2016) of the study.

**Fig 3:**
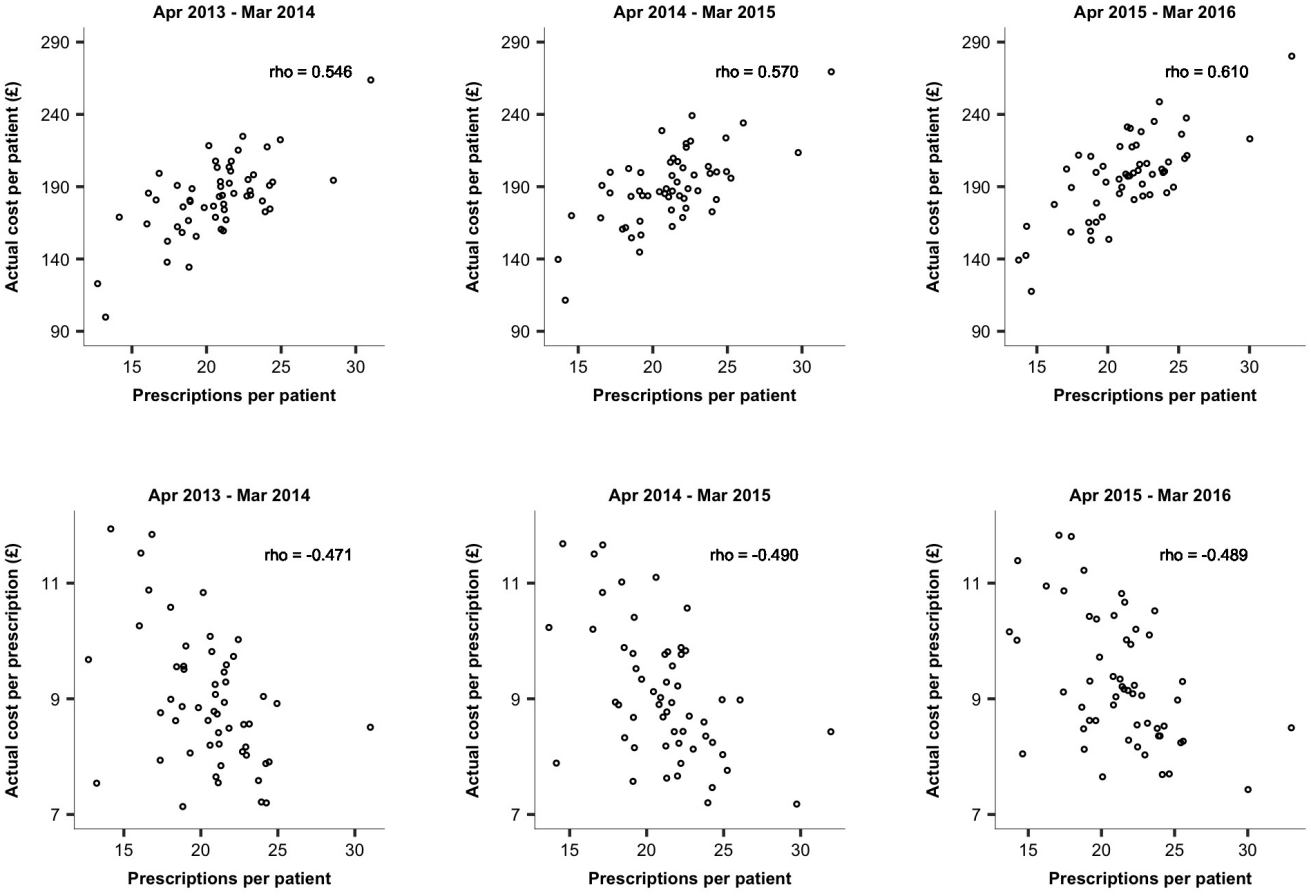
The relationship between standardized number of prescriptions and: A) the actual cost of prescribed medications per patient; B) the actual cost per prescription.

The distribution of costs through time appeared to show a similar trend to the prescribing rates. The moderate to strong association between prescription rates and actual costs of prescribed medications was reflected in the value of the Spearman's coefficient (Fig 3A). The *rho* was found to increase from 0.547 in Apr 2013 –Mar 2014 to 0.609 in Apr 2015 –Mar 2016. We also looked at the relationship between prescribing rates and the actual cost per prescription. We found those two measures to be moderately correlated (Fig 3B); the cost per prescription was shown to be lower for practices with higher rates of prescribing.

**Fig 4:**
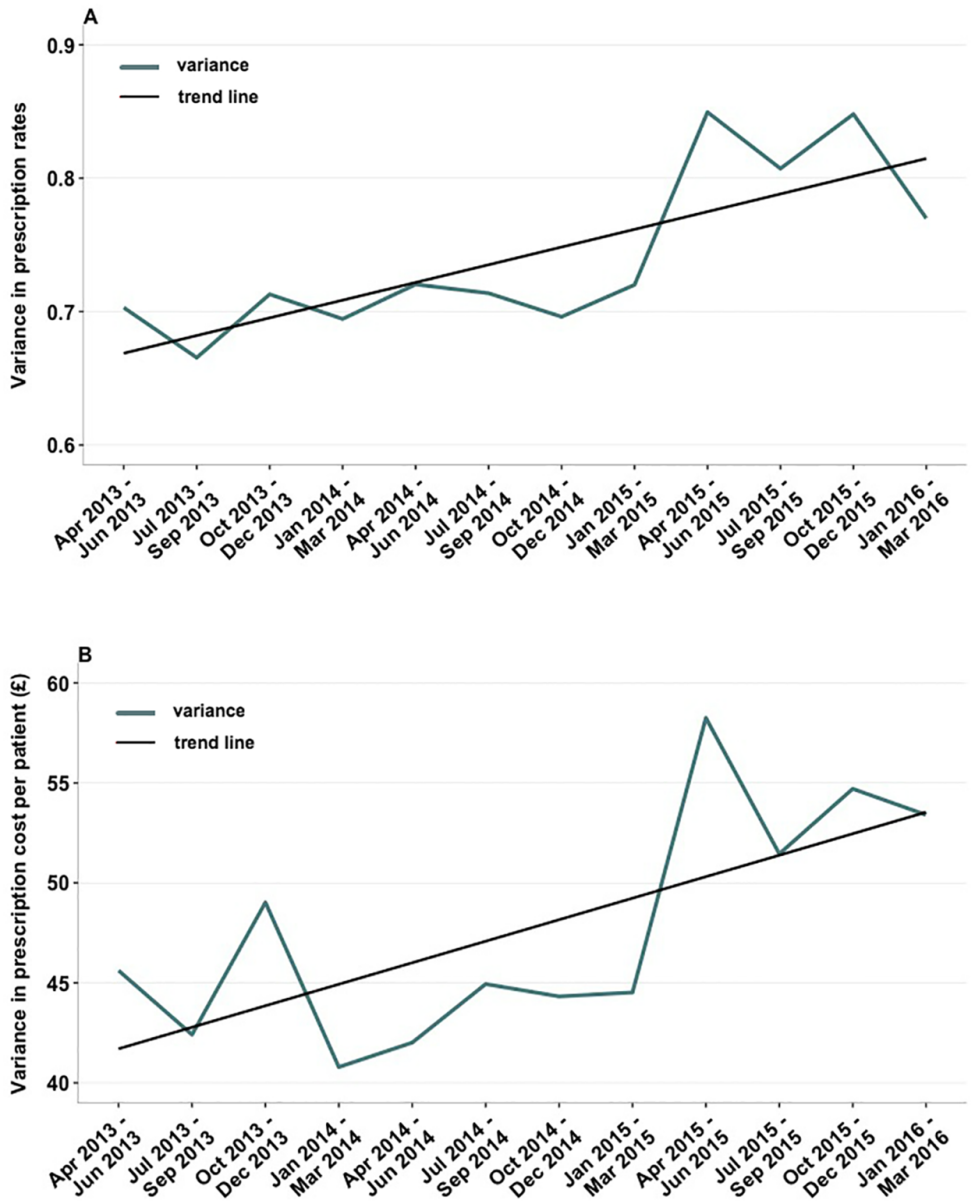
Temporal changes in variance calculated for: A) the number of prescriptions per patient; B) the actual prescription cost per patient for 55 investigated general practices. Black line represents the best-fit trend line for rates (A) and cost (B) of prescribing.

Our trend analysis showed that temporal changes in variability of prescribing rates and costs underwent an upward trend. Despite some temporal fluctuations in variance, the best fit line indicates that the value of σ^2^ for prescribing rates increased from £0.70 in Apr-Jun 2013 to σ^2^ = £0.77 in Jan-Mar 2016 (Fig 4). At the same time, the between-practice variability in prescribing costs increased from σ^2^ = £45.6 in Apr-Jun 2013 to σ^2^ = £53.4 in Jan-Mar 2016. The Mann–Kendall test confirmed a statistically significant upward trend in variability of GPs prescribing rates (*p* = 0.011) over the study duration.

Rural practices had a lower average number of prescriptions per patient than urban practices at all time points (Table 1). Over the period of investigation, the mean number of prescriptions per patient for rural practices rose by ~3.3% from 5.07, 95CI = [4.70,5.44] in Apr-Jun 2013 to 5.24, 95CI = [4.83,5.64] in Jan-Mar 2016 while urban practices reported a ~6.7% increase in average prescribing rate from 5.14, 95CI = [4.86,5.41] in Apr-Jun 2013 to 5.48, 95CI = [5.22,5.75] in Jan-Mar 2016. In all quarterly periods, the difference in the mean number of prescribed medications per patient between urban and rural practices was found statistically insignificant.

**Table 1:** Prescribing rates for rural and urban practices. T-test *p*-value refers to the significance level of differences in the mean number of prescribing rates between rural and urban practices for all considered time period. The *p*-value of F-test assesses the difference in variances in prescribing rates between rural and urban practices.

	Apr-Jun 2013	Jul-Sep 2013	Oct-Dec 2013	Jan-Mar 2014	Apr-Jun 2014	Jul-Sep 2014	Oct-Dec 2014	Jan-Mar 2015	Apr-Jun 2015	Jul-Sep 2015	Oct-Dec 2015	Jan-Mar 2016
*Rural*												
Mean	5.07	5.08	5.15	5.16	5.16	5.17	5.26	5.28	5.07	5.11	5.26	5.24
Variance	0.84	0.74	0.78	0.78	0.78	0.80	0.77	0.87	0.94	0.94	1.07	1.02
*Urban*												
Mean	5.14	5.19	5.23	5.23	5.34	5.24	5.39	5.33	5.31	5.43	5.50	5.48
Variance	0.62	0.62	0.68	0.65	0.68	0.67	0.65	0.62	0.79	0.69	0.68	0.58
F-test *p-value*	0.41	0.65	0.72	0.63	0.72	0.64	0.66	0.38	0.65	0.41	0.24	0.14
T-test *p-value*	0.77	0.64	0.72	0.78	0.44	0.77	0.58	0.83	0.35	0.21	0.36	0.32

Rural practices had a higher between practice variability than urban practices at all time points (Table 1). The variance for practices designated as rural grew from σ^2^ = 0.84 in Apr-Jun 2013 to σ^2^ = 1.02 in Jan-Mar 2016. This upward trend in variability was found statistically significant with *p* = 0.0032. Conversely, the variance for urban practices decreased from σ^2^ = 0.62 in Apr-Jun 2013 to σ^2^ = 0.58 in Jan-Mar 2016; however, this change was statistically insignificant (*p* = 0.54). At all studied time periods, F-test *p*-value showed no significant differences in variance in prescribing rates between rural and urban practices.

Practices situated in more deprived areas were found to have higher prescribing rates than those located in less deprived areas although this difference was not statistically significant in any of the considered quarterly periods (Table 2). The average number of prescriptions per patient in less deprived areas grew by ~7.5% from 5.0, 95CI = [4.38,5.61] in Apr-Jun 2013 to 5.37, 95CI = [4.76,5.98] in Jan-Mar 2016 while practices situated in more deprived areas reported a ~4.6% increase in mean prescribing rate from 5.14, 95CI = [4.92,5.38] in Apr-Jun 2013 to 5.38, 95CI = [5.13,5.62] in Jan-Mar 2016. The variability in prescribing rates for practices in less deprived areas was substantially higher than for practices in more deprived areas and this difference in variances was shown to be statistically significant for 8 quarterly periods (Apr 2013-Mar 2015) (Table 2).

**Table 2:** Prescribing rates for practices located in areas of different levels of socio-economic deprivation. T-test *p*-value refers to the significance level of differences in mean number of prescribing rates between practices from less and more deprived areas. The *p*-value of F-test assesses the difference in variances in prescribing rates between practices from less and more deprived areas. Asterisk: Statistically significant difference (*p* < 0.05) in variability in prescribing rates.

	Apr-Jun 2013	Jul-Sep 2013	Oct-Dec 2013	Jan-Mar 2014	Apr-Jun 2014	Jul-Sep 2014	Oct-Dec 2014	Jan-Mar 2015	Apr-Jun 2015	Jul-Sep 2015	Oct-Dec 2015	Jan-Mar 2016
*Less deprived areas*												
Mean	5.00	5.01	5.12	5.13	5.13	5.11	5.26	5.23	5.22	5.22	5.42	5.37
Variance	1.27	1.25	1.39	1.37	1.37	1.41	1.31	1.33	1.49	1.41	1.41	1.25
*More deprived areas*												
Mean	5.14	5.18	5.22	5.22	5.30	5.24	5.36	5.34	5.20	5.31	5.39	5.38
Variance	0.55	0.51	0.53	0.51	0.54	0.52	0.53	0.56	0.68	0.65	0.70	0.65
F-test *p-value*	0.046*	0.031*	0.022*	0.018*	0.027*	0.018*	0.032*	0.037*	0.065	0.066	0.096	0.117
T-test *p-value*	0.665	0.625	0.776	0.788	0.624	0.709	0.770	0.767	0.966	0.790	0.926	0.979

## Discussion

Over the period of investigation, the average between-practice variation in rates of prescribing was σ^2^ = 0.74, 95%CI = [0.71, 0.77]. The prescribing rates of individual practices ranged, on average, from 3.34, 95%CI = [3.26,3.42] to 8, 95%CI = [7.86,8.14] prescriptions per patient. At the same time, the average variance of prescribing costs was σ^2^ = £47.6, 95%CI = [£44.4, £50.8] with actual cost of prescribed medications per patient ranging, on average, from £27.2, 95%CI = [£26.1, £28.3] to £67.9, 95%CI = [£66.5, £69.3]. While it may be challenging to define what represents an appropriate rate or cost of prescribing, it is certainly difficult to justify large differences in prescribing between individual practices providing care to broadly similar groups of patients within a single healthcare system.

It is worth highlighting that both rates and costs of prescribing observed in the Northern Ireland Western Health and Social Care Trust were found to be higher than the rates and costs recorded in England. In 2015, an average of 18.6 items was dispensed in primary care for each patient registered with a GP practice in England [36] compared to 21.2 items per head issued in WHSCT. In England, the cost of prescribed items was roughly £157 per patient, £5 per patient higher than in 2014. In comparison, the average prescription cost per patient in WHSCT was £189.8, 95%CI = [182.9,196.7], ~£7.6 higher that in 2014. Despite higher average rates of prescribing per patient, the variation across England in the number of prescribed medications was higher than in WHSCT with the prescribing rates ranging from 9.5 to 33.3 items per head in 2015. At the same time, the number of items per patient issued in WHSCT ranged from 13.7 to 31.7 [36].

Since no demographic data was published alongside the GP prescribing data for WHSCT, we could not estimate the effect of demographics of patient population on variation in prescribing rates. Previous studies however showed that demographic characteristics of patient population did not fully explain prescribing behaviour of GPs [21]. Among the factors related to the varying prescription activity, age of patients was most often factored into analyses of variation [37], although age alone did not account for enough variation to develop an accurate model for predicting prescribing rates [38]. It was shown that age and gender accounted for approximately 25% of variation [39,40] and additional demographic characteristics (e.g. mortality rates) up to 51% [41].

Our study shows differences in both prescribing rates and between practice variation in prescribing between rural and urban practices. The mean number of prescribed items was higher in urban practices than in rural practices. The reasons for this are unclear and were beyond the scope of the present study. However, possible explanations include differing patient populations in rural and urban areas, differences in practice organisation and workflow, as well as differences in characteristics of general practitioners such as training, background, and age. Our results appear consistent with previous studies. For instance, in Scotland, lower levels of prescribing of antidepressants were found for practices in rural areas while higher rates were observed for urban practices [18]. In addition, lower rates of prescribing of psychotropic drugs were reported by rural/small town practices in Denmark [19].

Our results indicate higher levels of prescribing for practices located in more deprived areas of the Western Health and Social Care Trust and lower levels for practices from less deprived areas. Furthermore, the differences in variances of prescribing rates given different levels of local deprivation were found statistically significant for 8 quarterly periods. So far, several studies have demonstrated that socio-economic deprivation can influence prescription rates for some medications, such as antidepressants and lipid-lowering drugs. In England, the difference in the number of prescriptions between the bottom 1% and top 1% areas by deprivation was 20% [42,43].

In addition, we found that the variability in prescribing rates underwent a statistically significant upward trend reflecting larger deviations of prescribing rates of individual practices from the mean prescribing rate. This can be related to the changes in socio-economic and demographic characteristics of patient populations but we also cannot exclude possibility that these growing deviations may reflect growing differences in quality of care leading to, in fact, avoidable increase in prescribing costs. However, higher variability does not necessarily imply lower quality practice. It therefore requires further inspection to determine if the patient populations associated with specific GP practices are different and have different needs.

A moderate (Apr 2013-Mar 2015) to strong (Apr 2015-Mar 2016) relationship was observed between prescription rates and actual costs of prescribing; a higher cost of prescribed medications per patient was associated with a higher number of issued items per patient. The differences in pharmaceutical costs observed for the practices with similar prescription rates might be related to the type of prescribed drugs e.g. the cost of one pack of Amiodarone (100mg tablets) is £2.21 whereas for a pack of Allopurinol (100mg tablets), we have to pay over £35. The differences in prescription costs in practices with similar prescribing rates may also be associated with the medication choice i.e. a generic vs. brand name drug. There is evidence that inefficient prescribing by GPs increases NHS costs by hundreds of millions of pounds every year [21]. Of course, there can be legitimate reasons why patients require brand name drugs. However, our data do not allow us to examine the appropriateness of such decisions. We also found the number of items per patient to be negatively correlated with the actual cost per item i.e. the cost per prescription was shown to be higher for practices with lower rates of prescribing. It suggests that practices that prescribe more items per head appear to prescribe cheaper drugs.

We believe that the identification of outlier practices i.e. practices with higher or lower prescribing rates than the calculated outlier cut-off values may act as an important consideration when deciding which practices may benefit from interventions to alter prescribing behaviour of GPs [44]. That is, there might be greater merit in engaging with individual practices where prescribing rates appeared significantly higher or lower than average. The identification of such practices could reduce the time, effort, and cost of any intervention. However, we are aware that a statistical outlier in terms of prescribing rates is not equivalent to inappropriate practice and therefore, further analysis would be required to assess if higher/lower rates that outlier cut off values can be explained by characteristics of patient populations (e.g. age, ethnicity) or practice features (e.g. age, training of general practitioners).

The main limitation of our study results from its design. Our analysis was conducted to investigate the variability patterns and change in prescribing rates and costs, but due to data unavailability, we were not able to examine how the differences in patient or provider factors may affect variation in prescribing. Business Services Organisation in Northern Ireland does not provide free and open access to data sets related to demographic characteristics of patient population and practice features at the level of the GP practice. We believe that when such data becomes available, further investigation of characteristics of practices and patient populations in the Western Health and Social Care Trust may shed more light on other factors contributing to variations in GPs prescribing. This can help us to better address potential inappropriateness and inefficiency of prescribing.

In conclusion, our study provided information on variability patterns and temporal changes in rates and cost of prescribing in Western Health and Social Care Trust. We showed that practice setting and socio-economic deprivation account for some of the between-practice variation in prescribing. We suggest that optimisation of prescribing could be enhanced by conducting appropriate clinical interventions when other factors contributing to prescribing variation are identified. These interventions could include educational initiatives and feedback during which GP practices would be informed about their own frequency of prescribing relative to the mean prescribing of other practices. The prescribing behaviour of GPs could also be altered by comparing their past performance to clearly defined professional standards/targets. The quality improvement initiatives including normative feedback proved to be effective in decreasing variability in prescribing in the past [45].

## Supporting information

S1 Table The variance in prescribing rates with and without outlier practices. (PDF)
