# Supplementary material for: Prescriptive variability of drugs by general practitioners
Source: PLoS One. 2018 Feb 20;13(2):e0189599. doi: 10.1371/journal.pone.0189599 (PMC5819764; doi:10.1371/journal.pone.0189599)
Supplement: S1 Table — (PDF) [file pone.0189599.s001.pdf]

S1 Table. The variance in prescribing rates for the data set with and without outlier practices.

	Apr-Jun 2013	Jul-Sep 2013	Oct-Dec 2013	Jan-Mar 2014	Apr-Jun 2014	Jul-Sep 2014	Oct-Dec 2014	Jan-Mar 2015	Apr-Jun 2015	Jul-Sep 2015	Oct-Dec 2015	Jan-Mar 2016
With outlier practices												
σ^2 (£)	0.70	0.67	0.71	0.69	0.72	0.71	0.70	0.72	0.85	0.81	0.85	0.77
Without outlier practices												
σ^2 (£)	0.52	0.39	0.59	0.43	0.45	0.44	0.49	0.50	0.60	0.58	0.59	0.56
